# Curved Foldable Tailored Fiber Reinforcements for Moldless Customized Bio-Composite Structures. Proof of Concept: Biomimetic NFRP Stools

**DOI:** 10.3390/polym12092000

**Published:** 2020-09-02

**Authors:** Gabriel Rihaczek, Maximilian Klammer, Okan Başnak, Jan Petrš, Benjamin Grisin, Hanaa Dahy, Stefan Carosella, Peter Middendorf

**Affiliations:** 1 ITECH Masters Program, University of Stuttgart, Keplerstr. 11, 70174 Stuttgart, Germany; gabriel.rihaczek@gmx.de (G.R.); klammax@gmail.com (M.K.); okan1basnak@gmail.com (O.B.); 2 Department of Bio-based Materials and Materials Cycles in Architecture (BioMat) at Institute of Building Structures and Structural Design (ITKE), University of Stuttgart, Keplerstrasse 11, 70174 Stuttgart, Germany; hanaa.dahy@itke.uni-stuttgart.de; 3 Institute of Aircraft Design (IFB), University of Stuttgart, Pfaffenwaldring 31, 70569 Stuttgart, Germany; grisin@ifb.uni-stuttgart.de (B.G.); stefan.carosella@ifb.uni-stuttgart.de (S.C.); peter.middendorf@ifb.uni-stuttgart.de (P.M.); 4 Faculty of Engineering, Department of Architecture (FEDA), Ain Shams University, Cairo 11517, Egypt

**Keywords:** stiffness gradient, curved folding, digital fabrication, tailored fiber placement, bio-composites, moldless, natural fiber-reinforced polymers, NFRP

## Abstract

Fiber Reinforced Polymers (FRPs) are increasingly popular building materials, mainly because of their high strength to weight ratio. Despite these beneficial properties, these composites are often fabricated in standardized mass production. This research aims to eliminate costly molds in order to simplify the fabrication and allow for a higher degree of customization. Complex three-dimensional shapes were instead achieved by a flat reinforcement, which was resin infused and curved folded into a spatial object before hardening. Structural stability was gained through geometries with closed cross-sections. To enable this, the resource-saving additive fabrication technique of tailored fiber placement (TFP) was chosen. This method allowed for precise fibers’ deposition, making a programmed anisotropic behavior of the material possible. Principles regarding the fiber placement were transferred from a biological role-model. Five functional stools were produced as demonstrators to prove the functionality and advantages of the explained system. Partially bio-based materials were applied to fabricate the stool models of natural fiber-reinforced polymer composites (NFRP). A parametric design tool for the global design and fiber layout generation was developed. As a result, varieties of customized components can be produced without increasing the design and manufacturing effort.

## 1. Introduction

### 1.1. Fiber-Reinforced Polymers

Fiber Reinforced Polymers (FRPs) are increasingly popular building materials. This is mainly because of their high strength to weight ratio, but also high durability, stiffness, etc. [1]. As a composite material, a wide variety of matrix and reinforcement materials allow specifying the FRPs properties to its requirements. While fibers have their highest mechanical strength and stiffness in the fiber direction, the matrix transfers loads between the fibers and sets the final shape. By combining two or more base materials, the properties of the resulting composite material differ from the ones of the constituents. If the composite is well designed, certain properties better than the individual ones of the base materials can be achieved [2]. Furthermore, the lightweight and high stiffness of FRPs may reduce the need for concrete and metals in construction and allows for “another level of geometrical variations and integrated functionalities” [3].

Within this research a special interest lies in the use of natural fibers as a reinforcement material, thus creating a natural fiber-reinforced polymer (NFRP), also called bio-composite [3]. Although natural fibers were already used more than 100 years ago, the use was suspended due to the low cost and growing performance of synthetic fibers, while a renaissance in the use for technical applications has been recognized in recent years [4]. Research is conducted on the combination of natural fibers with a variety of matrix materials, such as polymers or concrete [5]. Different fabrication techniques such as vacuum infusion or other emerging techniques like fused deposition modeling (FDM) 3d printing can be used for the fabrication of natural fiber composites. For the example of FDM printing, the impact of the natural fiber reinforcement is directly linked to the used thermoplastic matrix material, while a multitude of processing parameters have to be taken into account [6].

It was shown that the replacement of synthetic fibers by renewable natural alternatives could contribute to lower CO_2_ emissions and help cope with the growing population [3]. This makes natural fibers very suitable for the future construction industry, which today accounts for 11% of the global energy-related CO_2_ emissions [7].

Natural fibers can be categorized according to their origin into lignocellulosic, animal, and mineral fibers, where lignocellulosic fibers can be categorized into wood fibers and non-wood fibers [8]. Hereby, non-wood fibers are an important alternative to wood-based fibers, due to a shortage of trees and increasing global demand [9]. Additionally, non-wood fibers are derived from plants that are fast-growing and only require months to mature, as compared to wood, which takes 20–30 years to mature [10].

There are multiple additional advantages of using bio-composites over petrochemical based composites, such as renewability, biodegradability, energy absorption, lower cost, lower weight, etc. [11]. Disadvantages include lower mechanical properties, higher moisture absorption, varying quality, lower durability, etc. [12]. However, in some cases, natural fibers can also exceed the mechanical properties of synthetic fibers, such as flax fibers in comparison to glass fibers in aligned discontinuous fiber composites [13]. 

Despite the described beneficial properties, NFRPs are difficult to fabricate with custom-oriented design methods. This is, in part, due to the time consuming, wasteful, and costly mold making process. This becomes an obstacle, especially for small quantities of parts and variations of similar shapes.

### 1.2. Curved Folding 

This research aims to eliminate molds in order to simplify the fabrication and allow for a higher degree of customization. Complex three-dimensional shapes are instead achieved by a flat reinforcement, which is resin infused and folded into a spatial object before hardening. Although this process could also be applied to straight folds, only curved folds were investigated. This is because, as opposed to straight folds, bending and subsequently curvature is induced to surfaces by the deformation of an adjacent curved crease. Curvature again can be used to achieve stiffness of the composite on a global scale.

While straight creases were investigated for centuries in traditional papercraft, such as Origami, the design with curved creases is still relatively unexplored. Early work in the field of curved folding was done by Josef Albers at the Bauhaus art school and dates back to the 1920s [14]. These explorations were focused on physical testing with paper and situated in an artistic realm. The computer scientist David A. Huffman expanded the field of known curved folding patterns significantly through analog as well as digital approaches [15]. In recent years, research in the fields of computational design and digital fabrication benefits the accessibility of the use of curved folding. There are approaches to large scale foldable structures, both with compliant and mechanical hinges [16]. Methods to construct rigid-foldable curved origami structures, as by Tomohiro Tachi [17], show the recent progress in the field of volumetric curved foldable structures. Inspiration for the shape of the prototypes was drawn from paper-craft by Richard Sweeney [18]. 

### 1.3. Tailored Fiber Placement

The additive fabrication technique of tailored fiber placement (TFP), which allows for the deposition of curvilinear fiber bundles, is chosen for the fabrication of the NFRP preforms. TFP is a common industrial embroidery-based process that uses a multi-head stitching machine that places continuous rovings along predefined curved paths within a plane on a flat textile base layer [19]. A near-net shape placement of materials is possible, meaning that there is little post-processing required after the fabrication. Therefore, it is potentially resource-saving in comparison to subtractively fitted isotropic reinforcement products such as fiber mats or weaves. 

The potential of curvilinear fiber placement lies in the production of variable axial (VA) preforms, resulting in a point-wise variation of the material properties such as stiffness and strength [20]. The geometrical freedom of TFP, as compared to other technologies, like automated fiber placement (AFP), allows for the formation of small radius loops and, therefore, a significant change in fiber density and stiffness [19], also enabling the definition of fold zones. This fact is used to overcome the disadvantage of flat preforms when compared to the possibilities of AFP, where pre-impregnated fiber tows can be laid out on a three dimensional form by pressing and bonding with a roller on a mold surface [21,22]. A drawback of TFP is that it is relatively slow when compared to AFP, as only a single bundle is laid out at a time, whereas a tape of pre-impregnated tow can be laid out at once using AFP. Additionally, the presence of stitching yarn leads to inconsistent fiber volume content, which has a negative effect on the performance of tailored fiber reinforced polymers (TFRPs) [19]. Fiber paths have to be defined according to a finite computing power [23]. Additionally, a deeper understanding of force distribution is needed to generate TFP layouts to compete against conventional production techniques [20]. 

The L1 Lightweight stool case study by the Leibniz-Institut für Polymerforschung Dresden has successfully proven that a topologically optimized TFP produced preform could provide a highly performing stool while using less material [24]. A drawback of this project is the complex mold setup.

Previous work by Aldinger and Margariti [25] concerning the moldless fabrication of FRP’s through TFP allows for the spatial formation of flat-produced preforms through elastic membrane contraction. However, it is limited to surface structures with continuous curvature as well as in size by the mechanical properties of existing membrane materials and machine sizes. The present contribution aims to enable a system with increased structural capacity due to the creation of closed cross-sections and curved folded edges. Additionally, shapes that cannot be achieved with classical molds can be fabricated.

**Figure 1:**
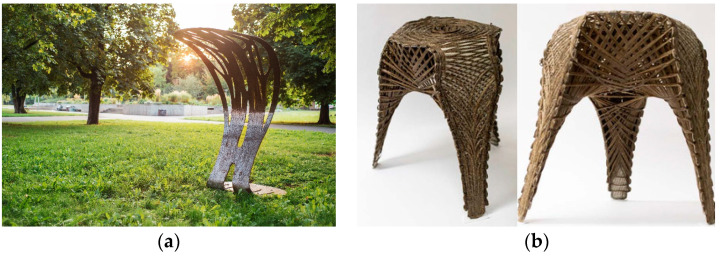
(**a**) Tailored biocomposite mockup by BioMat [26]; and, (**b**) “FlexFlax stool” [27].

The Tailored biocomposite mock-up (Figure 1a) developed by BioMat at ITKE/University of Stuttgart is a generatively designed single curved canopy structure, optimized in order to minimize the material usage [26]. Multiple designs were analyzed and the one with the lowest deformation was chosen. The focus of this project was to apply continuous flax fibers as a reinforcement agent of a biocomposite. There were three stages of the fabrication, namely TFP, mold preparation, and the vacuum-assisted molding process. Four layers of preforms with maximum dimensions of 1.0 × 1.4 m defined by the workspace of the machine were produced taking overlaps in between them into account. Additionally, a reusable mold with a waffle structure was produced. The preforms were then impregnated with epoxy resin in a closed vacuum-assisted process on the mold. After the structure was removed from the mold, additional reinforcement was added, and post-processing took place. 

The mock-up has successfully proven that TFP with flax fiber reinforcement can be used on a building scale, possibly resulting in precise shell or panel structures with controlled fiber orientation for architectural purposes. However, a mold was needed to connect the single preforms and to define the final shape. This means that the described fabrication procedure is suitable for the fabrication of a repeated shape but unsuitable for customized geometries, which the present research aims to enable. 

The “FlexFlax stool” (Figure 1b) was developed as part of the seminar Material Matter Lab IV at the University of Stuttgart, as well as this research project. This stool aimed to explore the potentials of combining TFP with coreless fiber winding to realize spatial structures without molds or frames. Four main fabrication steps were completed; the TFP of the preform, resin vacuum infusion of the preform, bending of the infused preform using a clamping rig and winding of continuous fibers onto the bent preform. The syntax layup for the coreless winding step consisted of a spine, bracing, and locking syntax. Flax fiber and epoxy resin were used for this project, as well as a glass fiber stitching base material. A typological study was performed on the bent preform surface to guide the TFP surface design. The final weight of the prototype was 1080 g while supporting an 80 kg person. 

Although an additional structural benefit can be drawn from winding onto a hardened and bent preform, the high performance may be counteracted by an additional labor-intensive fabrication step [27]. This means that higher structural performance could potentially be achieved while using the combination of TFP and fiber winding. However, fiber winding takes additional time and labor after the fabrication of the preform, representing a potential drawback.

Instead of stiffening the preform by a multi-method approach, the present research aims to integrate as much functionality into the preform as possible and create a sturdy structure by folding only. 

### 1.4. Scope

Within the scope of this research, the development of a continuous parametric workflow of curved folded NFRPs was achieved, covering all phases, from design to production. This presents a novel approach to designing TFP structures, where manual work is reduced to the minimum in order to fully exploit the benefits of computation and the customizable fabrication process. Additionally, the developed production workflow stands out against previously described references, because it allows for a forming process that is completely free of molds or scaffolds. A special interest lied in a variety of geometries, based on developable surfaces joined at curved intersections. The described approach is in line with the current trend in the construction industry from standardized towards customizable mass production of unique geometries. 

A study of a biological role-model was conducted to transfer knowledge regarding the anisotropic placement of fibers from nature into the design process. 

## 2. Materials and Methods

The development was embedded in a bottom-up design approach, where the design is the synthesis of multiple fields. Starting from the material system, the fabrication and forming of the NFRP were investigated, while taking geometrical and structural considerations into account. The used materials and developed design and fabrication methods are subsequently described in this section.

### 2.1. Programmed Anisotropy

In general, FRPs can be reinforced either with short chopped fibers or continuous fibers. However, FRPs with chopped fibers do not reach the same elevated structural efficiency of continuous fiber reinforcement. Common continuous fiber reinforcements are weavings of wrap and weft bundles at an angle of 90°. This results in a bidirectional reinforcement with similar stiffness in two directions. 

**Figure 2:**
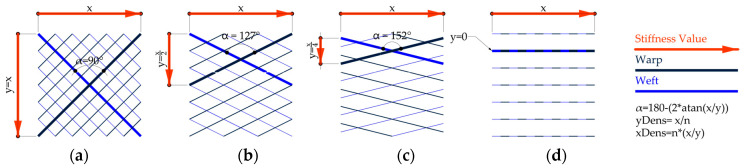
Reinforcement diagrid variations. Bidirectional(**a**), intermediate (**b-c**), unidirectional (**d**).

In Figure 2, warp and weft are represented in two separate layers. By changing the angle at the intersection points from 90° to other values, the stiffness according to x and y can be altered. In between a bidirectional (Figure 2a) and a unidirectional (Figure 2d) reinforcement are infinite intermediate steps (Figure 2b–c). While using this principle, the stiffness and, therefore, bending direction of a surface can be programmed by gradually changing the crossing angles. This method is applied to a grid-based fiber layout generation, as illustrated in Section 2.3.3.

### 2.2. Biomimetics

Making an object foldable requires the definition of curves along which the material deforms. The exoskeleton of arthropods was analyzed as a biological role-model in order to understand how to utilize the potentials of the anisotropic material the best. The exoskeleton consists of the inner cellular epidermis and the outer non-cellular cuticula. The cuticula itself is built up in three layers. The outermost and very thin epicuticle, the exocuticle, and the innermost endocuticle. Both the exocuticle and endocuticle contain chitin fibers, while the exocuticle also contains other constituents and it is harder [28]. The exocuticle is formed of cylindrical rigid plates called sclerites. Because these plates are not able to bend, the locomotory freedom of these invertebrates is ensured by membranous regions or sutures in between the sclerites. At these junctions, the exoskeleton is thin and flexible, because it lacks the exocuticle. Additionally, the material is folded here and additional surface area is provided when the joints are bent [29].

**Figure 3:**
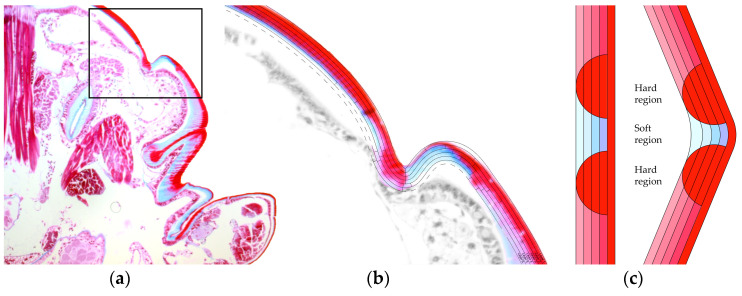
A section through the exoskeleton of a flea in detail in (**a**) and magnified in (**b**). These two photographs were taken by the authors at the Department of Evolutionary Biology of Invertebrates at the University of Tübingen. Abstracted representation of the hinge zone in flat and folded state (**c**). The reinforced edge is shown as semi-circles along the fold, while the colors show the differentiation between the harder exocuticle (red) and softer endocuticle (blue), where the deformation takes place.

A section of a flea was analyzed under the microscope. The exocuticle is stained red, while the endocuticle is stained blue (Figure 3a). A closer look (Figure 3b) allows to identify how the shell is built up. It can be observed that the stiffness changes in a zone between two shell elements. Two rigid elements are connected with a flexible part between them. In addition, a thicker edge of the plates can be recognized. This indicates that the flexible plates are reinforced at the edge in order to keep the shape even under bending and maintain the articulated connection. In conclusion, two principles are abstracted. Rigid parts are connected with a hinge zone instead of a thin line. The edge of the hinge zone is stiffer than the hinge zone itself. Figure 3c shows an abstracted representation of a fold zone in section. 

### 2.3. Workflow—Design

A design to production workflow was developed in order to prove the feasibility of the intended bio-composite shaping process. The workflow included both design and engineering-related aspects that were integrated to simplify the process and make it more accessible, also to inexperienced users. The workflow starting from the prototyping until the creation of fabrication files was handled in Rhinoceros^®^—Grasshopper^®^ Computational Modelling Software (Robert McNeel and Associates, Seattle, WA, USA). The design and fabrication processes were materialized on a furniture scale. A stool was chosen as a prototypical demonstrator. This is because a stool is similar to a structural system that needs to support a high load when compared to its weight, while also fulfilling aesthetic requirements. It was aimed at fabricating stools that can support an adult person with a weight of at least 100 kg.

#### 2.3.1. Prototyping

Paper models are a fast method of designing folding structures on a small scale. In an empirical process, parts can be easily added or cut away in order to refine the shape. Even though paper is an isotropic material with a theoretical zero thickness, the characteristics can be transferred onto a larger scale. 

**Figure 4:**
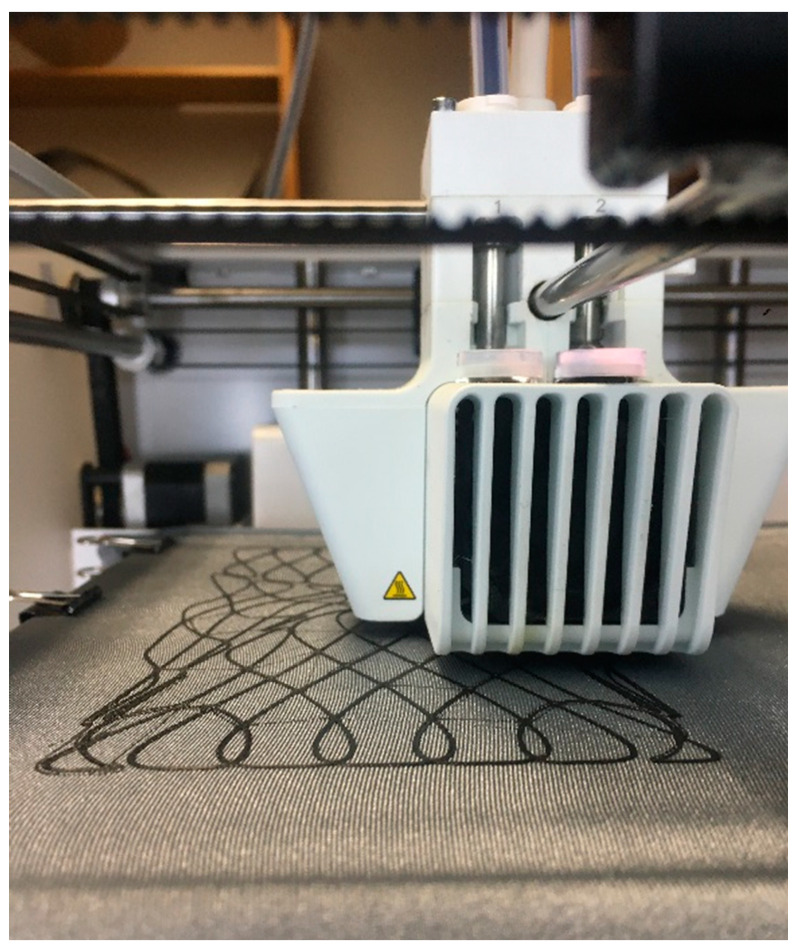
Three-dimensional (3D) printing onto the textile.

A three-dimensional (3D) printer was used by the authors to allow faster prototyping with the possibility of exploring a wide range of patterns and design parameters. The extrusion of thermoplastic filaments onto stretched textiles gave comparable properties to that of the final prototypes (Figure 4). In contrast to paper models, the anisotropy of TFP was represented through this method. An Ultimaker 3 printer (Ultimaker B.V., Utrecht, Nederland) was used and Ultimaker PLA filaments were deposited. The files were created using the Silkworm Plugin for Rhino Grasshopper^®^, with simple modifications in post-processing.

#### 2.3.2. Form Generation

A point-symmetrical design was chosen. The flat layout was based on a polygon with a minimum of three sides. There was one curved fold per side and the edge was roughly offset from the crease outwards. In addition to the point-symmetry, there was an axis-symmetry of each leg. This allowed the folded legs to be connected on the inside to form a closed and thus stronger cross-section. In contrast to the Tailored biocomposite mockup that was developed by BioMat [26] and “Tailoring Self-Formation” [25], the folding creates not only two-dimensional surface structures but also structural depth for a sturdier self-supporting structure.

**Figure 5:**
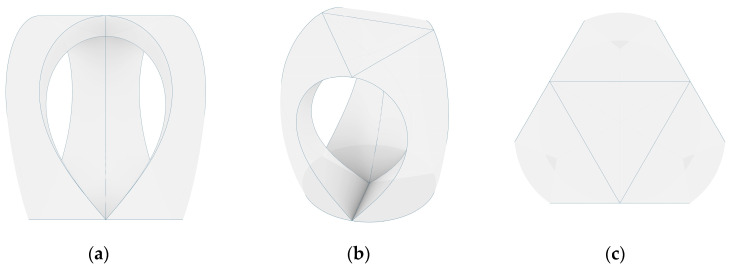
Digital model of a stool with three legs in a front view (**a**), perspective (**b**) and top view (**c**).

The initial design was based on curves in two-dimensional space. There are two reasons why a two-dimensional (2D) to 3D transformation was essential to the design process. The first is to visualize the three-dimensional structure (Figure 5) for the designer, making iterative paper folding studies obsolete. The second is to allow for the open edges of the design to be correctly set and touch when the part is folded.

A geometrical modeling approach for both the 2D and 3D states was chosen. Because the dimensions of the used textile do not change during the folding, the lengths can be transferred from the 2D to the 3d structure and vice-versa. There are four input parameters; the leg count, leg length, seat radius (*R_Seat_)*, and base radius (*R_Base_*).

**Figure 6:**
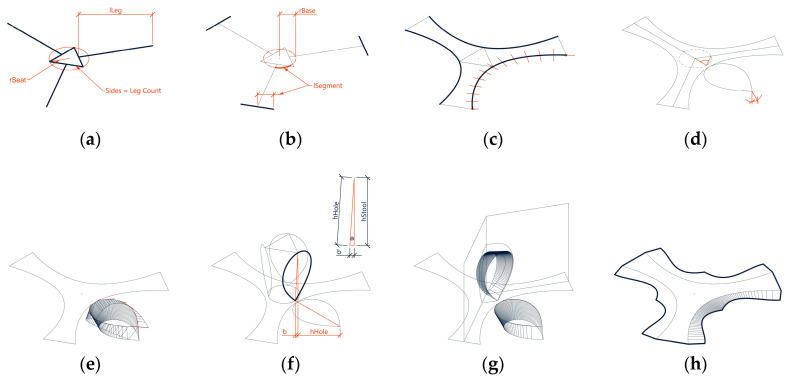
Geometric form-finding for a stool with three legs. Definition of the base polygon (**a**), base radius determination (**b**), construction of base arc-curve (**c**), construction of fully folded spatial curve (**d**), construction of ruled surface (**e**), inclination of the spatial curve towards base circle center (**f**), orientation of ruled surface along the inclined spatial curve (**g**), mapping of ruling lengths (**h**).

- First, a polygon with one side per leg and the seat radius as the outer radius was constructed. Lines perpendicular to the mid-point of each polygon edge with the leg length were added (Figure 6a).
- Another circle with the base radius was constructed and split into twice the amount of legs segments. The length of one segment defined the length of the outer edges (Figure 6b).
- Based on the endpoints of these edges and one corner of the polygon, a three-point arc-curve was constructed. The normal and tangent vectors of this curve were extracted (Figure 6c).
- Because the transformation of this arc-curve matches with that of an elastica curve, an elastica curve with identical length and midpoint, but touching ends was drawn. The latter curve defined the fully folded spatial curve. The height of this curve (*h_Elastica_*) was measured. Folding angles were evaluated based on the curve vectors and the final folding angle defined by the base radius (Figure 6d).
- Ruled surfaces along the curves were constructed for different folding angles (Figure 6e).
- Because the base radius was set at a lower value than the seat radius, the edges of the stool were slightly inclined towards the base circle center (Figure 6f). Equations (1) and (3) define the angle of inclination (α). Equation (2) computed the total height of the stool.
  b = |R_Base_ − R_Seat_|, (1)
  h = (h_Elastica_^2^ − b^2^) ^1/2^, (2)
  α = π/2 − arccos(b/ h_Elastica_), (3)
- The ruled surface is oriented along the inclined spatial elastica curve. The ruling lengths were measured when the surface is intersected with the two adjacent mirror planes (Figure 6g). The ruled surfaces were also constructed between the spatial elastica curves according to lengths in the two-dimensional layout. The seat as one flat polygonal surface connects the midpoints of the curves. With a total of seven individual surfaces for a three-leg design, the spatial state was fully defined.
- By mapping the ruling lengths onto the two-dimensional layout, the outer curve was drawn (Figure 6h).

#### 2.3.3. Pattern Generation

**Figure 7:**
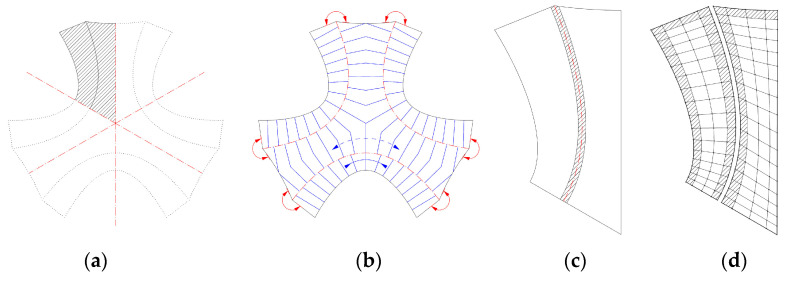

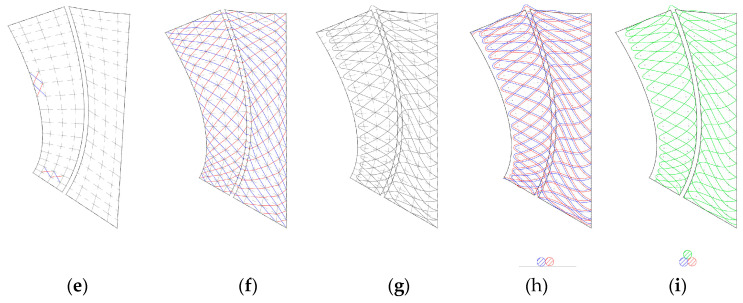
Process of pattern generation. Part with basic patter (**a**), definition of edges (**b**), definition of hinge zone (**c**), quad division (**d**), definition of diagonal lines (**e**), conversion to interpolated NURBS curves (**f**), definition of end tangents by edge type (**g**), determination of base fiber layout (**h**), determination of second layer pattern (**i**).

The fiber layout for the TFP was based on the two-dimensional outline curves. The pattern was only designed for one part of the symmetrical layout (Figure 7a). First, the surface edges were defined as a rigid edge, a hinged edge, or an open edge (Figure 7b). According to the biological role-model, a hinge zone is defined along the curved crease (Figure 7c). The layout was generated following the logic of programmed anisotropy, which is illustrated in this section. The area was divided into quads (Figure 7d) and every quad contains two diagonal lines (Figure 7e). The aspect ratio and size of the quads determined the direction and strength of the reinforcement, as well as the stiffness of the structure. For the generation of the quad cell dimensions, multiple requirements were taken into account, as shown in Figure 7d:

- The quads were compressed along the bending direction of the elastica curve to allow for the bending.
- Along the open edges, the quads were compressed to be aligned with the edge and thereby reinforce it.
- Along the curved creases, the quads were compressed, so that the fibers cross the crease at a low angle to maintain the minimum bending radius of the fiber bundles and prevent fibers from breaking.
- In the middle of the surface, the quads were larger because the forces were lower. In between, the size was interpolated.

The quad edge vertices were connected to form the fiber directions as soon as the quad sizes were defined based on the explained factors. These polylines (Figure 7f) were converted into interpolated NURBS curves with end tangents defined by the edge type (Figure 7g). 

The design was intended to highlight the materials anisotropy and structural differentiation. Instead of evenly distributing fibers over the whole surface, higher structural capacity was instead achieved by repeating the stitching pattern with slightly offset curves. A pyramid cross-section was built up along the basic pattern to gain height. Two fiber bundles were placed directly next to each other (Figure 7h) and one on top in the middle of both (Figure 7i). To do this, the base layer was offset from the rowing radius from the main direction.

The pyramidal layering logic was also used to implement the principle that was developed based on the biological role-model. Only two out of the three curves of the pyramid crossed the crease in order to enable the structure to fold along the pre-defined curves. The top fiber was instead placed tangential to the crease, without crossing it (Figure 7i). This resulted in a reinforced edge in relation to the crease thickness.

**Figure 8:**
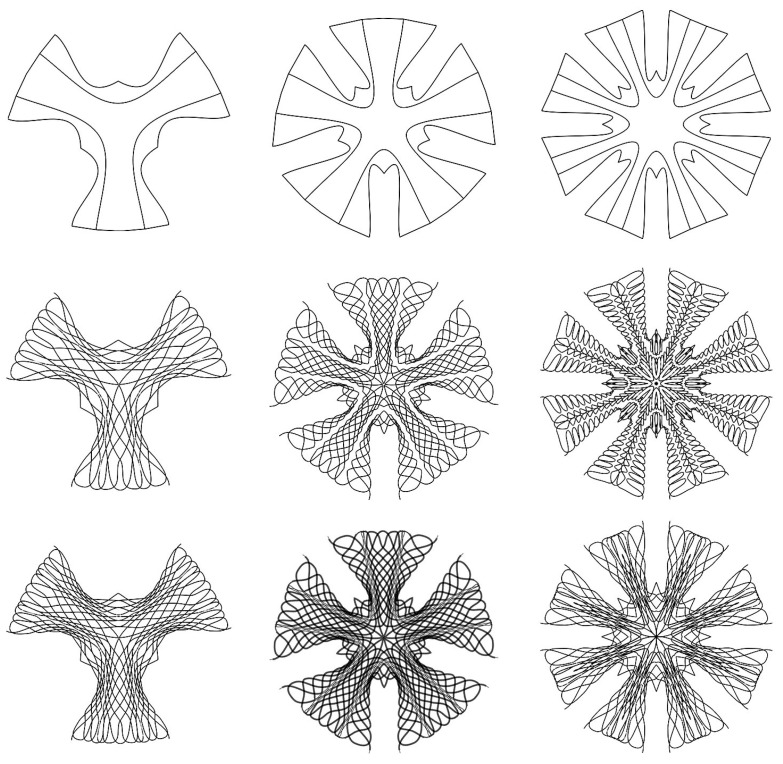
Parametrically generated options of the fiber layout for three, five, and eight legs with different fiber orientations and lengths.

To sum up, a fiber pattern was developed that solved multiple problems of the hinge zones, edges, main vertical stress direction, and the design intent. In the case of the stool, the pattern was relatively even since there were multiple load cases to resist. The parametrically generated fiber layout can be applied to various designs of stools (Figure 8). However, a more optimized solution for a predominant load case needs to be further explored for use in an architectural application.

### 2.4. Workflow—Fabrication

#### 2.4.1. Flax Fiber

The selected fibrous material for the TFP process was a commercially available non-twisted flax fiber with 2400 tex provided by Groupe Depestele, Bourguebus, France. Flax fiber is chosen, because of its availability in Europe as well as for its high strength of 500–900 MPa, which exceeds that of many other natural fibers [4]. The fiber volume fraction of flax fiber is lower than, for example, carbon fibers. Using vacuum infusion, a fiber volume content of 30% can be achieved for flax fibers, while a content of 50% can be achieved for carbon fiber reinforced samples [30]. For the fabrication of the stools, this value for flax fiber was set as a target and criterion in order to evaluate the resin impregnation at 1 bar. However, a fiber-content higher than 30% by weight was desired.

#### 2.4.2. Tailored Fiber Placement

The fiber bundles were laid with a stitch width of 3,5 mm, resulting in a circular cross-section. The stitch length was constant at 7 mm, but it could be adapted to the curvature to speed up the fabrication process. Polyester sewing yarn was used with Amann Serafil 120/2 for the upper and Amann Serafil 200/2 for the under thread. A white cotton textile was chosen as a base material. A Tajima machine with four stitching heads was used for the prototypes, with a maximum work envelope of three by one meter, at the IFB institute of the University of Stuttgart. A speed average of 500–600 stitches per minute was set.

#### 2.4.3. Resin Vacuum Infusion

**Figure 9:**
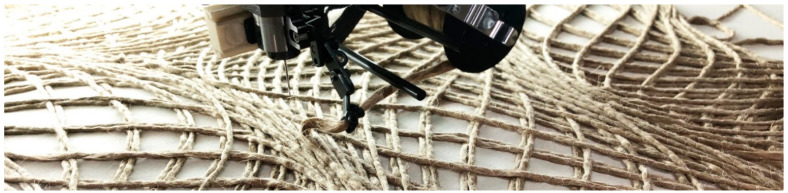
Tailored fiber placement process.

**Figure 10:**
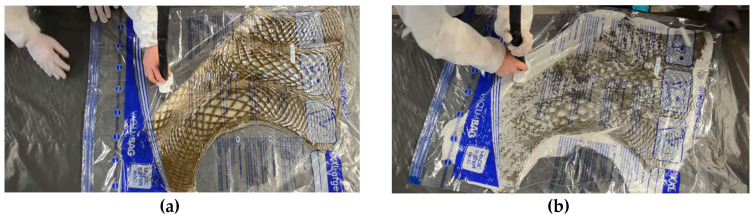
Resin vacuum infusion process (**a**), removal of excessive resin with a sacrificial material (**b**).

The preforms that were produced using the TFP (Figure 9) were first cut out along the outline. The preforms were then placed in a vacuum bag and infused with commercially available epoxy resin (three parts EPIKOTE Resin MGS RIMR 235, and one part EPIKURE Curing Agent RIMH 237, (Figure 10a). The epoxy resin was provided by Hexion Stuttgart GmbH, Stuttgart, Germany. A common vacuum bag for clothing was used and the preforms were each folded in halves due to the relatively large size of the preform pieces. The amount of resin that was needed for the infusion was approximated as 70% of the final composite weight, according to the previously set fiber weight ratio of 30% or resin weight ratio of 70%. Therefore, the resin was computed as 2.3 times the weight of the preform. Because this estimation was made to make sure that all parts of the preform are infused equally, there was excessive resin. This excess resin was removed with a sacrificial fleece before the preform was removed from the vacuum bag (Figure 10b). 

Up to this point of the research, the main intention of the infusion process was to keep it as simple and accessible as possible, while using commonly available materials. However, it is also apparent that the amount of resin could be lowered for further prototypes, eliminating the need for the removal of excess resin. To save more resin and, at the same time, not compromise the structural properties, a more extensive study on the ratios has to be conducted in the future, also taking the chosen textile material into account. 

#### 2.4.4. Forming

**Figure 11:**
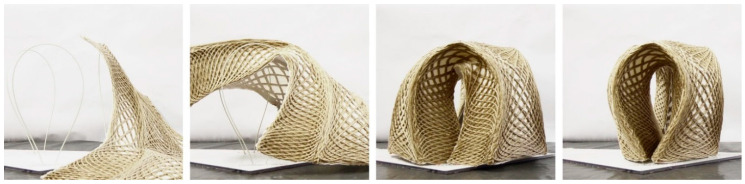
Process of placing a preform on bent scaffolding rods.

Two techniques of moldless forming were explored. The first option was forming with a minimal scaffolding (Figure 11). For this, glass fiber rods with a diameter of 2mm were bent and fixed on a base. The infused preform was placed on the bent rods in such a way that the crease curves matched the elastic curves of the bent rods. After curing, the rods were removed for the use in a different configuration. This technique was used for the first prototype. Although it worked well, the designs were still limited to elastic curves. 

**Figure 12:**
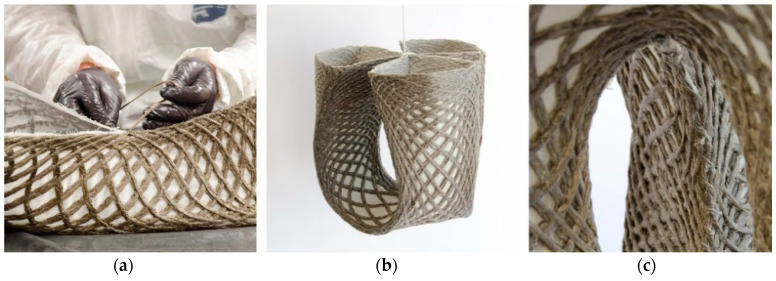
Stitching of the legs (**a**) and forming of preform under self-weight (**b**). Closeup photography of the stitched legs after curing (**c**).

The second option stands out because no custom scaffolding was required. Instead, the shape was formed by the self-weight of the infused preform. The shape was retained by the connection of the open ends of the preform through stitching (Figure 12a). When the ends were completely stitched together by a simple zigzag stitch, the preform was turned around and suspended until the resin had cured (Figure 12b). The same flax fiber as for the TFP was used as a thread, as well as a common stitching needle.

#### 2.4.5. Patching

**Figure 13:**
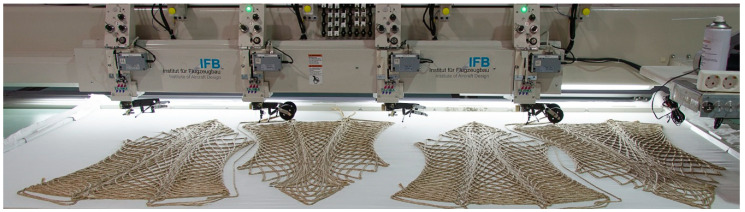
Fabrication of multiple legs on a Tajima machine.

**Figure 14:**
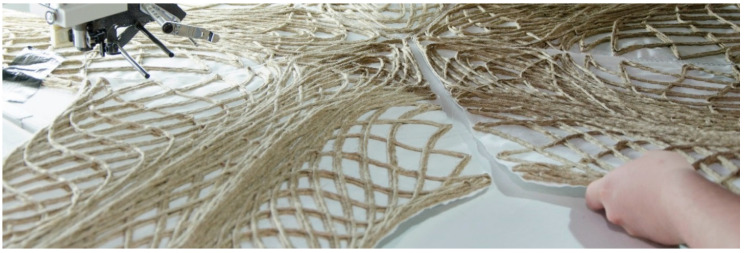
Placing the last fifth of the third prototype on the TFP machine for the connection.

The method of patching and connecting on the machine was developed to overcome the size limitation of the machine. Instead of fabricating the stool in one continuous part, the individual legs were first separately produced. This gave the advantage that multiple stitching heads were used in parallel, speeding up the whole fabrication process (Figure 13). Although the used Tajima machine had four stitching heads, only two were running in parallel due to the size of the parts. The prefabricated legs were aligned on the machine and connected with a centrally placed fiber path within the work envelope (Figure 14). This technique was utilized for the third prototype, which otherwise would have exceeded the fabrication size limitations.

## 3. Results

Utilizing the workflow that was developed as part of this research and explained in Section 2, five stools were produced in total. The finished prototypes show both aesthetic and functional qualities. An adult person of at least 100 kg can sit on any of them, thereby meeting the initially set aim. This means that prototypes one and five as the lightest stools support a weight 83 times higher than their self-weight. This was tested physically; mechanical testing needs to be conducted in future work in order to assess the maximum load-bearing capacity. Additionally, the stools are sturdy enough to be handled and stored and they offer a proof-of-concept that can be similarly upscaled for larger structural elements for architectural applications. By placing more fiber bundles on top of each other and filling the empty areas as well, the strength of the parts could be significantly increased for architectural applications. 

**Figure 15:**
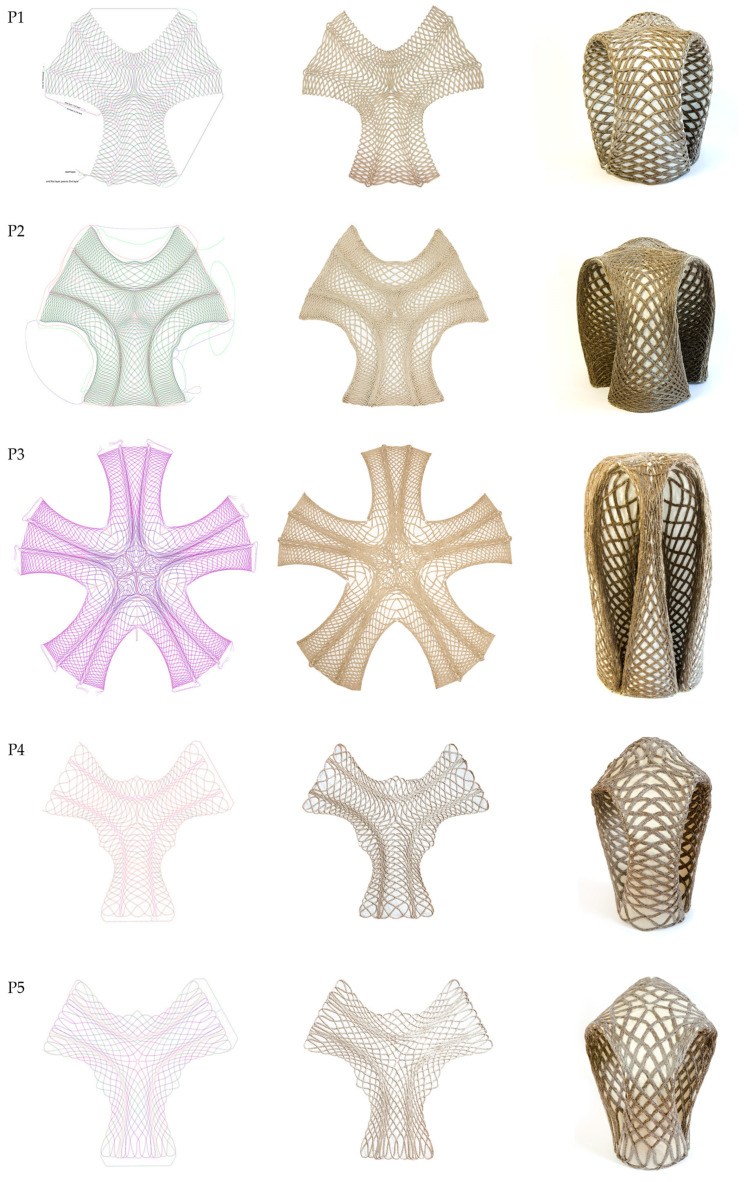
Column 1: Digital fiber layout; column 2: fabricated preforms; column 3: final stools of prototypes 1–5; prototypes 1–5 in rows 1–5.

The prototype one was formed by the placement on bent rods. Because its shape did not allow achieving closed legs, the ends were not stitched together. As a result, there was a higher deflection when a physical sitting-test through a person weighing 100 kg took place in comparison to the other prototypes, tested with the same stated conditions. The technique of forming under self-weight and stitching was applied for prototype two to five. While this worked well for the shape of prototype two, this process was not suitable for the third prototype due to its increased weight and elongated shape. Although the correct shape was approximated, the surface was wrinkled in contrast to the evenly curved surfaces of the smaller prototypes. Because the amount of resin per gram of textile was unknown, the total resin weight was used to compute the fiber weight fraction and fiber volume fraction. Consequently, the computed ratios are slightly lower than in reality. However, the intended fiber volume fraction of 30% was achieved for two prototypes. This shows that, by further improving the fabrication process, values of more than 30% could be achieved for all geometries. For all prototypes, the preform is infused homogeneously. A comparison of five prototypes is further described in Table 1 and illustrated in Figure 15.

### Kinetic Structures

At the current state, the resin system allowed a non-reversible forming process. By making the forming process reversible, the use of curved folded NFRPs could be extended. Not only static structures but also compliant kinetic parts e.g., as shading systems or temporary spatial divisions could be designed. Compliant kinetic mechanisms have multiple advantages over conventional rigid-body mechanisms. Fewer parts are required for movement, resulting in a more economic system with simplified manufacturing and assembly, as well as reduced maintenance [31].

Initial tests were also performed by applying liquid wood glue at the hinge before resin vacuum infusion was applied in order to understand the possibilities and problems of a kinetic hinge. This prevented the infusion with resin. The wood glue was washed out when the resin has been hardened, to re-enable the flexibility of the hinge. 

**Figure 16:**
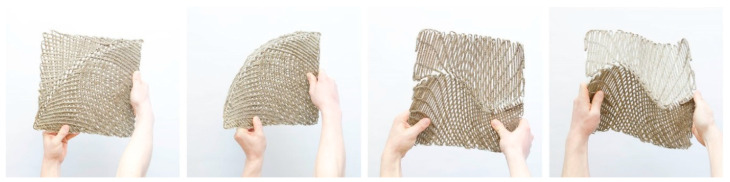
Sample 1 (left) and sample 2 (right) with compliant curved hinges.

In the samples that are shown in Figure 16, the effect of fiber direction and crease type on the flexibility was tested. It was noted that the flexibility remained higher when the fibers cross at a low angle to the crease and stayed lower when the fibers cross perpendicular. In the first sample, this was tested on a single curved crease. When one part of the piece got actuated, the other deformed accordingly. In the second sample, it was tested on a more complex crease with an inflection point. The sample behaved similarly to the first one and it was comparable to the same folding pattern.

Although these initial tests were promising, the fibers in the hinge remained exposed when the wood glue was removed. For a more durable hinge, thermoplastic polyurethane (TPU) is currently being explored. 

## 4. Discussion

The design and fabrication of moldless spatial NFRPs through curved folding is a suitable technique, which is made accessible by a comprehensive parametric workflow. Because the TFP process itself only allows one to create two-dimensional parts, the folding along material embedded compliant hinge zones was proven to be a viable solution to create geometrically stable structures. It is shown that the design was not limited to a certain shape, but it is rather versatile by being parametrically generated. A common difficulty in the use of TFP was how to translate a shape into a fiber layout, which was solved as illustrated within the process. The digital design process had facilitated the fabrication process through the settled parametrically defined dimensions. It was also shown that larger components can be produced by patching without the need for larger TFP machines. However, the main limitation in the use of TFP for curved foldable structures and larger architectural components lie generally in the relatively slow production speed, as compared to the fabrication of e.g., fiber weaves. The produced prototypes required 1.5 to 4 h of machine time, depending on the amount of placed fibers. Whereas, diverse ways to increase the production speed were separately explored, like through parallel production of legs as in prototype three as well as through the usage of a higher linear density fiber, or simply through using an upgraded machine with more stitching heads. 

Two of the composite components, the flax fiber, and the cotton textile, are made from annually renewable bio-based plant-resources. They are unhazardous and haptically pleasing, which makes them very suitable for furniture and architectural building elements, with which humans directly interact with. The highest potential for further optimization of the material system lies in the substitution of the petro-based epoxy resin with a plant-based resin variation. Bio resins could potentially complement the flax fibers and cotton textile to reach a 100% green biocomposite, while, on the other hand, the use of TPU could enable reaching kinetic NFRP structures. While there are many options for potential substitutes, changing the resin system would also mean that the performance is affected. Therefore, the choice for the resin system needs to be carefully evaluated based on the given requirements. The time of usage, type of usage, and location are linked to this evaluation. Additionally, for bio-resins specifically, factors like land use and biodegradation over time and for thermoplastic resins factors like the maximal use temperatures, need to be included in the evaluation.

**Figure 17:**
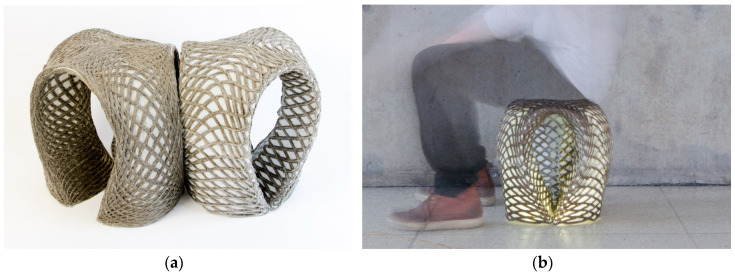
Possible connection of two components (**a**), prototype in use with integrated lighting (**b**).

Although the prototypes were designed as furniture, the basic intention is to benchmark this development towards upscaling it to reach an architectural realm in the near future. With the integration of connection detailing, the developed prototypes could then be assembled into a larger structure (Figure 17a), especially considering that each unit can take loads around 100 kg (ca. 1000 N). Furthermore, for the following developments, software for structural analysis is intended to be integrated into the digital design workflow in order to generate more optimized fiber layouts, depending on the forces types and flow. This became a necessity on large scale structures, where components need to be locally adapted, depending on specific load cases. A destructive load test has not been performed up to the date of publication but will be essential for the further development of structural components. 

A range of shapes that are based on developable surfaces joined along curved creases can be designed and fabricated using the developed workflow. Despite that only one family of curved foldable shapes was investigated within this research, this does not display the full range of possibilities. Although design limitations were set by the forming process, many more designs based on either elastica curves for the minimal scaffolding forming or catenary curves for the self-weight forming can be explored and materialized. The light-weight and structured translucent appearance of the components leads to novel aesthetical qualities (Figure 17b), enabling new ways of architectural expressions, coupling the benefits towards future-oriented sustainable architecture.

## Figures and Tables

**Table 1:** Quantitative comparison of the five prototypes.

		P 1	P 2	P 3	P 4	P 5
**Height**	cm	36	38	73	44	44
**Diameter**	cm	32	32	34	26	26
**Fiber length**	m	162	315	565	132	129
**Stitch count**	-	23,142	45,000	80,714	18,857	18,428
**Preform weight**	g	460	790	1640	416	400
**Fiber weight**	g	389	756	1356	317	310
**Textile weight**	g	71	34	284	99	90
**Resin weight**	g	669	1296	2676	864	780
**Total weight**	g	1200	2120	4600	1280	1180
**Fiber weight fraction**	%	32	36	30	25	26
**Fiber volume fraction**	%	31	31	28	22	24
